# Perception, Knowledge, Indications, and Future Prospects of Point-of-Care Ultrasound Among Medical Students in Saudi Arabia

**DOI:** 10.7759/cureus.57704

**Published:** 2024-04-06

**Authors:** Saeed M Nassar, Sarah A Almubrik, Lama Alyahya, Mohammed Alshalan, Hussain M Alhashem

**Affiliations:** 1 Department of Emergency Medicine, King Saud University Medical City, Riyadh, SAU; 2 Department of Emergency Medicine, King Saud University, Riyadh, SAU

**Keywords:** pocus (point of care ultrasound), teaching in emergency medicine, emergency and trauma radiology, medical student training, medical education curriculum

## Abstract

Introduction

Point-of-care ultrasound (POCUS) has become integral across medical specialties globally, addressing clinical queries, guiding procedures, and bridging the gap between physical examination and advanced imaging. Early ultrasound training for medical students enhances clinical decision-making and reduces diagnostic errors.

Aims

To evaluate the knowledge and attitude of senior medical students towards POCUS and to assess knowledge gaps and difficulties encountered by senior medical students to assist in the development of future curricula.

Methodology

This is an observational, cross-sectional approach to evaluate knowledge, attitude, and practice of POCUS among senior medical students in the Kingdom of Saudi Arabia. The study was conducted from January to September 2023. An electronic questionnaire was distributed through online platforms utilizing medical school databases across various regions. The survey encompassed sociodemographics, training methods, diagnostic indications, and participants' self-reported proficiency and attitudes toward POCUS. The data was chiefly collected using the Likert scale. Descriptive statistics were used to describe the quantitative and categorical variables. Bivariate and multivariate analyses were used to examine correlations.

Results

A total of 359 senior medical students completed the survey. Most responders were females (57.9%) with the predominating age group being ≤ 24 years (83.6%). The students predominantly were from the Central region of Saudi Arabia (75.5%). Ultrasound training varied among responders; 31.5% received formal courses (median duration: two hours) and 23.4% informal courses (median duration: four hours). Around 17.3% practiced POCUS self-teaching (median duration: four hours). A total of 3.6% had formal POCUS accreditation. A gargantuan 82.2% never used POCUS in their attached hospital for a variety of reasons. Multivariable logistic binary regression analysis showed a positive correlation between students' self-teaching of POCUS and their perceived difficulty performing an ultrasound examination for patients in daily practice.

Discussion

A comparable study was done at King Saud bin Abdulaziz University for Health Sciences (KSAU-HS) in 2022 surveying 229 senior medical students by Rajendram et al. In their study, 21.4% completed formal courses and 12.7% took informal courses. While many students in our study were not exposed to POCUS (82.2%), KSAU-HS reported a higher percentage reaching 94.8%. A study by Russel et al. demonstrated more than half of 154 surveyed medical schools in the United States have implemented POCUS into their students’ curriculum.

Conclusion

POCUS stands as a valuable skill that can enhance the educational journey of undergraduate medical students. Considering that a significant number of participants haven't yet taken formal medical school courses suggests a lack of awareness about its significance in the medical field. Offering additional courses with practical components could enhance the proficiency, confidence, and outlook of medical students toward POCUS.

## Introduction

Point-of-care ultrasound (POCUS) has played a role in nearly all medical specialties and is rising globally. POCUS has been used at the patient's bedside to answer specific clinical questions, assess treatment, and guide different procedures [1]. POCUS effectively fills the gap between physical examination and more advanced imaging like CT or MRI. Therein, POCUS exhibits its useful applications in trauma surveys for intraperitoneal free fluid, assessment of volume status, and much more [2].

That being said, ultrasound has become an important subject to be taught and has already been integrated into many medical schools' curricula. Early ultrasound training enhances students' clinical decision-making abilities, increases their comfort level, and improves their competency while utilizing ultrasonography in clinical rotations [3,4]. By enhancing students' capacity to acquire precise ultrasound images, early ultrasound instruction for medical students may reduce future diagnostic mistakes [5].

Growth in clinical use of ultrasound necessitates the integration of POCUS into a medical school curriculum. Some studies have shown that ultrasound training enhances medical students' overall knowledge base, improves the accuracy of their physical examination skills, and improves their comprehension of relevant anatomy and physiology [6]. Ultrasound training in postgraduate trainees who need to improve in the basic knowledge and uses of ultrasounds, either due to not enough exposure or lack of a well-designed curriculum in medical school, can lead to avoidable difficulties in any training program they desire in the future.

It appears that medical students need to be able to perform and learn how to improve skills in the use of introductory POCUS before graduation and should become familiar with an emergency ultrasound application and be exposed to additional emergency ultrasound exams throughout their clerkship to increase their ability and confidence as interns, especially those working in underserved areas with limited access to Emergency Medicine (EM) residents or EM-trained attending physicians [7]. There is ample evidence that students can learn ultrasound knowledge and skills and that they enjoy and desire more ultrasound training in medical school [8].

## Materials and methods

A quantitative, observational, cross-sectional study was conducted from January 2023 to September 2023. Stratified random sampling was carried out through an electronic questionnaire; the link was distributed through the databases of colleges of medicine in Riyadh, Jeddah, Eastern Province, West and Southern Province. Participants included in the analysis were above 18 and senior medical students from the chosen colleges.

Participants filled out a written consent at the beginning of the questionnaire, informing them about the purpose of the study and how their participation contributes to the population. Participants were also informed that their participation was voluntary and that whenever they wanted to stop answering, they had the right to. Confidentiality and autonomy were assured by giving each participant a number, which will be used for analysis only. The researchers appreciated the time the participants gave them, and no reward or incentive was given to those who answered the questionnaire.

An electronic questionnaire from various studies [9-24], consisting of four sections, was distributed through the databases of the colleges of medicine in all the provinces of Saudi Arabia. For each participant, the following will be filled: the first section included sociodemographics (age, gender, university), and the second section contained questions about training, accreditation, and the usage of POCUS. The third section assessed the applicability of 15 diagnostic indications for POCUS. The fourth section investigated the ability of participants to perform POCUS along with a self-report of participants' knowledge of the principles of POCUS-related ultrasonography. In the fourth section, participants were asked to consider their attitude towards training in POCUS and the obstacles to training.

Data was analyzed by using the Statistical Package for the Social Sciences (IBM SPSS Statistics for Windows, IBM Corp., Version 24.0, Armonk, NY). Descriptive statistics (median, frequencies, and percentages) were used to describe the quantitative and categorical variables. Bivariate statistical analysis was carried out using the Chi-square statistical test based on the type of study and outcome variables. The bivariate Pearson's correlation test assessed the correlations between the metric-measured variables. The multivariable binary logistic regression analysis was conducted to determine the statistically significant predictors for students' odds of formal POCUS education, self-learning POCUS, attendance of free courses, and difficulty performing ultrasound. The association between students' measured predictor variables with analyzed outcomes was expressed as a multivariable-adjusted odds ratio with 95% confidence intervals. A p-value of <0.05 was used to report the statistical significance.

## Results

A total of 495 medical students submitted the online questionnaire. However, 359 (78%) senior medical students had completed it and fit the inclusion criteria. The resulting descriptive analysis of the students' sociodemographic characteristics is detailed in Table 1. Most respondents were female (57.9%), and the remainder were males (42.1%). Most of the students were ≤24 years (83.6%), and 16.4% were ≥25 years. The universities the respondents originated from were divided into respective regions to project regional variability. The regions of teaching/training were listed in descending order by response rate: The Central region comprised 75.5% and garnered the majority, the Eastern region comprised 10%, the Southern region comprised 7.5%, the Western region comprised 5.8%, and finally the Northern region comprised 1.1% of total respondents.

**Table 1:** Descriptive Analysis of Students’ Sociodemographic Characteristics, N=359 KSAU-HS: King Saud bin Abdulaziz University for Health Sciences; CR: central region; WR: western region; NR: northern region; ER: eastern region; SR: southern region

	Frequency	Percentage
Sex		
Female	208	57.9
Male	151	42.1
Age groups		
≤ 24 years	300	83.6
≥ 25 years	59	16.4
Location		
Eastern Region	36	10
Western Region	21	5.8
Northern Region	4	1.1
Southern Region	27	7.5
Central Region	271	75.5
Universities		
Al Baha University (WR)	8	2.2
Al-Faisal University (CR)	8	2.2
Al-Maarefa University (CR)	13	3.6
Dar Al Uloom University (CR)	12	3.3
Imam Abdulrahman bin Faisal University (ER)	12	3.3
Imam Muhammed bin Saud Islamic University (CR)	32	8.9
Jazan University (SR)	3	0.8
Jeddah University (WR)	2	0.6
Jouf University (NR)	1	0.3
King Abdulaziz University (WR)	3	0.8
King Faisal University (ER)	24	6.7
King Khalid University (SR)	17	4.7
King Saud University (CR)	118	32.9
KSAU-HS - Jeddah (WR)	3	0.8
KSAU-HS - Riyadh (CR)	76	21.2
Najran University (SR)	7	1.9
Northern Border University (NR)	1	0.3
Princess Norah University (CR)	6	1.7
Qassim University (CR)	6	1.7
Tabuk University (NR)	2	0.6
Taibah University (WR)	4	1.1
Umm Al-Qura University (WR)	1	0.3

The students also answered questions detailing the varying forms of ultrasound training they've received (Table 2). They were asked to indicate in dichotomy (No/Yes) whether they had already chosen their preferred medical specialty. A total of 53.6% of them had already chosen their desired specialty, and when asked in further detail, 41.8% of the students preferred to specialize in a medical specialty, while 12% preferred a surgical specialty. Surprisingly, the majority remained undecided (46.2%).

**Table 2:** Descriptive Analysis of Students’ Previous Point-of-Care Ultrasound Experiences and Training, N=359 POCUS: point-of-care ultrasound

	Frequency	Percentage
Have you chosen the field you would like to specialize in?		
No	166	46.2
Yes	193	53.8
Broadly, what specialty do you prefer?		
Medical specialty	150	41.8
Surgical specialty	43	12
Undecided	166	46.2
Did you receive/are you receiving formal training (i.e. a structured comprehensive course for all students organized by the College of Medicine) in POCUS during medical school?		
No	246	68.5
Yes	113	31.5
The number of formal hours training in POCUS during medical school per course, median (IQR)		2 (4.5)
Did you receive/are you receiving informal training (i.e. organized by students or doctors independent of the College of Medicine) in POCUS during medical school?		
No	275	76.6
Yes	84	23.4
The number of Informal hours training in POCUS during medical school per course, median (IQR)		4 (6)
Have you attended (free) courses on POCUS outside of medical school?		
No	335	93.3
Yes	24	6.7
The number of Free training hours in POCU during medical school per course, median (IQR)		5 (4)
If you had previous (FREE) training, did any of these include hands-on scanning?		
No	348	96.9
Yes	11	3.1
Have you ever paid to attend courses on POCUS outside of medical school?		
No	348	96.9
Yes	11	3.1
The number of Paid training hours in POCUS during medical school per course, median (IQR)		5.5 (6.5)
If you had purchased paid courses, did any of these include hands-on scanning?		
No	348	96.9
Yes	11	3.1
Are you teaching yourself POCUS? (e.g. via books or online resources)		
No	297	82.7
Yes	62	17.3
The number of hours self-training on POCUS, median (IQR)		4 (7.9)
What sources do you use for self-learning?		
YouTube	15	42.9
Books	9	25.7
Websites	12	34.3
Lectures	2	5.7
Other online medical sources	11	31.4

In addition, the survey explored the different ways students could register for POCUS courses and the nature of said courses. The analysis showed that 31.5% of the students had received formal structured and comprehensive training courses at their medical colleges on POCUS while studying at college, and the median duration (in days) for those formal training courses was equal to two hours with an interquartile range (IQR) of 4.5 hours. Also, the findings yielded that only 23.4% of the students had previously received informal training courses on POCUS provided by students and other sources independent of their medical school. Those informal courses' median duration days are four hours with an IQR of six hours.

Moreover, a small subset of students (6.7%) had attended free POCUS training courses during their training as medical students. The median duration for those free courses was five hours, with an IQR of four hours. Moreover, the analysis findings showed that a minute percentage (3.1%) of students in these free POCUS training courses received hands-on skills training on scanning patients. Interestingly, a mere 3.1% of students had previously purchased a paid POCUS training course with a median duration of 5.5 hours and an IQR of 6.5 hours. These paid courses also included hands-on training sessions.

Furthermore, the survey aimed at describing different ways students learned POCUS independently. This shows that 17.3% of the students are practicing POCUS self-teaching and learning, and the median duration of self-teaching sessions was equal to four hours with an IQR of 7.9 hours. When these students were asked to indicate the sources for self-instructed POCUS, 42.9% of the students mentioned YouTube videos, 25.7% learned from Books, 34.3% from online websites, and 5.7% from lecture notes, and 31.4% of them learned from other miscellaneous online medical sources dedicated for POCUS and radiological information.

**Figure 1:**
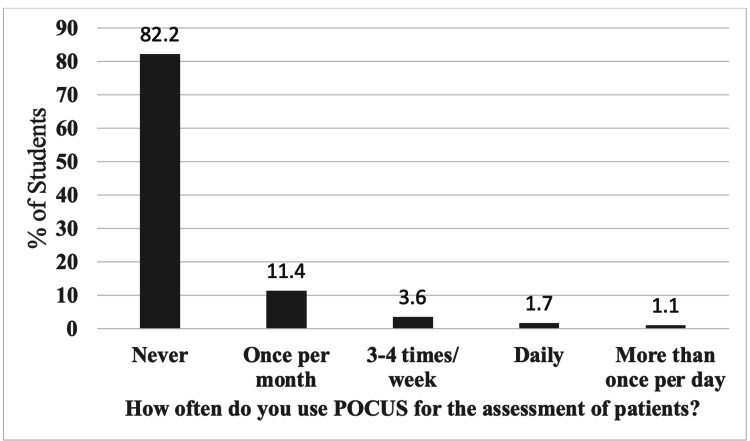
Frequency of Point-of-Care Ultrasound Use Among Senior Medical Students POCUS: point-of-care ultrasound

Also, the survey investigated whether or not these students had obtained any form of accreditation and a quantitative assessment of their POCUS experience (Table 3). A mere 3.6% of students had a formal accreditation, of which 3.3% of them had formal accredited training on echocardiography as well. To highlight the rate at which students encounter POCUS daily in their attached hospital, a significant majority (82.2%) of them never used POCUS, and 17.8% used it in their training. Of those that used POCUS, 11.4% of them used POCUS to assess patients once per month, 3.6% of them used POCUS three to four times per week, and 1.7% of them used POCUS daily, but a minuscule 1.1% of the students use POCUS more than once per day to assess their patients (Figure 1).

**Table 3:** Descriptive Analysis of Students’ POCUS Accreditation and Quantitative Personal Experience, N=359 POCUS: point-of-care ultrasound

	Frequency	Percentage
Do you have any formal accreditation in POCUS? (Accreditation: is a formal certificate of eligibility of practice)		
No	346	96.4
Yes	13	3.6
Do you have any formal accreditation in Echocardiography? (Accreditation: is a formal certificate of eligibility of practice)		
No	347	96.7
Yes	12	3.3
How often do you use POCUS for the assessment of patients?		
Never	295	82.2
Once per month	41	11.4
3-4 times/week	13	3.6
Daily	6	1.7
More than once per day	4	1.1
If you have never used POCUS for assessment of patients, why?		
No theoretical experience	103	28.7
No time	8	2.2
No training	85	23.7
Not my job	89	24.8
Radiology support is inadequate	18	5
How many times have you encountered a situation when you would have wanted to perform an ultrasound examination but were not able to do so because of the lack of supervisor/teacher (and not because of the lack of ultrasound machines)?		
Never	173	48.3
Rarely/a few times	105	29.2
Many times	55	15.3
Most of the time	26	7.2

Further, the students who had never used POCUS to assess patients were asked to indicate the reasons behind not using it during the assessment of patients and the findings showed that 28.7% of them had no experience with POCUS at all. An interestingly low percentage of students (2.2%) believed that they didn't have enough time for POCUS assessment. A significant percentage thought they lacked training (23.7%), while an astonishing 24.8% believed assessing the patients with the POCUS ultrasound is unnecessary. A significant minority (5%) of students felt that they didn't have sufficient support from their medical radiology departments in the attached hospital.

The survey assessed the student's perception of the applicability of POCUS skills to diagnose varying pathologies. This was conducted using a 1-5 Likert-like agreement scale, and the findings are detailed in Table 4. Most students agreed with the applicability of POCUS in identifying abdominal free fluid. Other important diagnoses included deep vein thrombosis (DVT), pneumothoraxes, thyroid masses, pleural effusions, and hepatomegaly. Surprisingly, in their insight, the least applicability of POCUS skills was assigned to interstitial lung disease, followed by lung consolidation, systolic heart failure, and cardiogenic shock. Unsurprisingly, these findings were synonymous with students' self-rated skillfulness in picking up different medical diagnoses in physical examination (Table 5), which was conducted in the same manner. To further assess the perceived theoretical knowledge/capability in physical examination, students were asked to assess their competency in applying POCUS to identify various diagnoses in the same manner as above; the responses again reflected their perceived usefulness and competency in physical examination (Table 6).

**Table 4:** Descriptive Analysis of Students’ Perceptions of the Applicability of Point-of-Care Ultrasound Skills for Diagnostic Purposes DVT: deep venous thrombosis; JVP: jugular venous pressure

	Mean	SD	Mean Rank
Determining the height of the JVP	3.63	1.29	17
Identifying pleural effusion	3.97	1.14	6
Identifying pneumothorax	4	1.21	4
Identifying interstitial lung disease	3.59	1.24	18
Identifying lung consolidation	3.74	1.21	15
Identifying hepatomegaly	3.96	1.13	7
Identifying splenomegaly	3.95	1.13	9
Identifying ascites/free fluid	4.07	1.1	1
Identifying abscess/cellulitis	3.92	1.15	11
Identifying DVT	4.05	1.12	2
Identifying abdominal aortic aneurysm	3.94	1.18	10
Identifying ectopic pregnancy	3.96	1.14	8
Identifying cardiogenic shock	3.79	1.22	14
Identifying thyroid masses	3.99	1.16	5
Intra-abdominal hemorrhage	4.01	1.16	3
Identifying renal masses	3.83	1.14	13
Identifying systolic heart failure	3.72	1.26	16
Identifying pericardial effusion	3.91	1.22	12

**Table 5:** Descriptive Analysis of Students' Self-Rated Skillfulness In-Patient Physical Examinations DVT: deep venous thrombosis; JVP: jugular venous pressure

	Mean	SD	Mean Rank
Determining the height of the JVP	3.1	1.31	10
Identifying pleural effusion	3.26	1.22	8
Identifying pneumothorax	3.49	1.18	3
Identifying interstitial lung disease	2.7	1.1	18
Identifying lung consolidation	3.26	1.23	9
Identifying hepatomegaly	3.58	1.2	2
Identifying splenomegaly	3.48	1.21	5
Identifying ascites/free fluid	3.7	1.16	1
Identifying abscess/cellulitis	3.36	1.19	7
Identifying DVT	3.39	1.14	6
Identifying abdominal aortic aneurysm	2.9	1.2	12
Identifying ectopic pregnancy	2.73	1.25	17
Identifying cardiogenic shock	2.76	1.15	16
Identifying thyroid masses	3.49	1.21	4
Intra-abdominal hemorrhage	2.94	1.22	11
Identifying renal masses	2.8	1.23	15
Identifying systolic heart failure	2.87	1.25	13
Identifying pericardial effusion	2.84	1.24	14

**Table 6:** Descriptive Analysis of Students' Self-Rated Skillfulness With Various Point-of-Care Ultrasound Procedures DVT: deep venous thrombosis; JVP: jugular venous pressure

	Mean	SD	Mean Rank
Determining the height of the JVP	2.13	1.12	17
Identifying pleural effusion	2.38	1.19	11
Identifying pneumothorax	2.45	1.2	8
Identifying interstitial lung disease	2.08	1.03	18
Identifying lung consolidation	2.28	1.18	14
Identifying hepatomegaly	2.55	1.26	4
Identifying splenomegaly	2.52	1.25	5
Identifying ascites/free fluid	2.64	1.32	1
Identifying abscess/cellulitis	2.37	1.22	13
Identifying DVT	2.6	1.28	3
Identifying abdominal aortic aneurysm	2.38	1.22	12
Identifying ectopic pregnancy	2.43	1.25	10
Identifying cardiogenic shock	2.14	1.07	16
Identifying thyroid masses	2.61	1.3	2
Intra-abdominal hemorrhage	2.51	1.27	6
Identifying renal masses	2.46	1.26	7
Identifying systolic heart failure	2.22	1.16	15
Identifying pericardial effusion	2.45	1.24	9

The bivariate Pearson's correlations method was used to assess the correlations between the students' measured POCUS perception (Table 7). The students' mean attitude toward improving bedside skills by learning body organ ultrasonography correlated positively and significantly with their attitudes toward POCUS training and learning benefits. Also, the students' mean attitude toward improving bedside skills via learning body organ ultrasonography correlated positively and significantly with their mean perceived skillfulness in physical examination and their perceived applicability of POCUS skills to patients' conditions and medical procedures. Moreover, the students' mean perceived self-rated proficiency with POCUS procedures had correlated positively with their mean measured attitude toward improving bedside skills via learning body organs ultrasonography (r=0.135, p-value<0.050), denoting that as the students perceived skillfulness with POCUS procedures tended to rise their mean attitude toward improving bedside skills via learning body organs ultrasonography score tended to increase incrementally too.

**Table 7:** Bivariate Correlations Between Students' Measured Perceptions About Point-of-Care Ultrasound Training and Patient Physical Examination **Correlation is significant at the 0.01 level (2-tailed). *Correlation is significant at the 0.05 level (2-tailed). POCUS: point-of-care ultrasound

	A	B	C	D	E
Attitude toward improving bedside skills via learning Body Organs Ultrasonography score (A)	1				
Attitudes and opinions about learning and training POCUS skills benefits score (B)	0.744**				
Physical examination skillfulness self-rating scale questionnaire score (C)	0.276**	0.317**			
Perceived applicability of POCUS to clinical procedures questionnaire score (D)	0.410**	0.511**	0.347**		
Self-rated knowledge of POCUS procedures questionnaire score (E)	0.092	0.083	0.359**	0.089	
Self-rated proficiency of POCUS procedures questionnaire score (F)	0.135*	0.123*	0.522**	0.095	0.718**

A multivariable logistic binary regression analysis was used to examine the association between students' self-teaching of POCUS with measured variables of interest (Table 8); this uncovered many key findings attributed to the behavior of medical students toward POCUS. The analysis showed that the students' sex and age did not correlate significantly with their odds of being a POCUS self-learner. Students who had already chosen their specialty were significantly more likely to be POCUS self-learners than those who stated they were undecided. It's important to note that as the students' self-rated POCUS proficiency tended to rise with one point on the Likert-like scale, their odds of being a POCUS Self-learner tended to rise. Also, the students who had previously attended a free or paid POCUS course and those who received formal, comprehensive POCUS training during college were found to be significantly more inclined to be POCUS self-learners. The students' mean perceived difficulty performing the POCUS procedures also correlated positively with their odds of being a POCUS self-learner, showing that a challenging skill encouraged self-development.

**Table 8:** Multivariable Logistic Binary Regression Analysis of Students' Odds of Self-teaching/Learning Point-of-Care Ultrasound POCUS: point-of-care ultrasound

	Multivariate adjusted odds ratio (OR)	95% C.I. for OR		
		Lower	Upper	p-value
Sex = male vs. female	0.891	0.463	1.715	0.731
Age (years)	0.997	0.812	1.226	0.98
Had already chosen their preferred specialty as of yet	2.022	1.062	3.85	0.032
Rate of POCUS use for the assessment of patients at workplace	1.375	0.943	2.004	0.098
Mean self-rated POCUS proficiency score	1.445	1.072	1.948	0.016
Had previously attended free courses	3.337	1.225	9.09	0.018
Had formal comprehensive POCUS training during college	2.124	1.137	3.969	0.018
Perceived difficulty performing an ultrasound examination for patients in daily practice	1.371	1.028	1.828	0.032
Had previously purchased POCUS courses outside school	6.081	1.268	29.17	0.024
Constant	0.018			0.1

## Discussion

POCUS is an advanced portable diagnostic modality used at the patient's bedside for various purposes. POCUS use has significantly increased in the last decade among different medical specialties to better diagnose, assess, and manage patients. It's a relatively flexible and available radiological modality with no significant risk to the patient. Medical schools can implement courses to help medical students better understand anatomy and disease identification. Early POCUS training can help empower medical students to shape their careers and make better clinical decisions owing to better knowledge with enhanced physical exam skills when supplemented by ultrasonography.

As Low as Reasonably Achievable (ALARA) is the basic principle that should be implemented for protection against radiation generated by different radiological modalities; ultrasound is no exception in regards to its acoustic output. A multitude of precautions can be found in the literature that can be followed: Limitations of transducer dwell time and total scanning time, using the least amount of power to generate a diagnostic image, and monitoring the mechanical and thermal indices requisite during imaging [25,26]. Even though POCUS is used without restrictions by most medical practitioners and no adverse effects have been demonstrated or confirmed in humans, the ALARA principle using POCUS should be implemented, particularly when conducted on the eye, lung, and fetus [27].

In this study, 31.5% of senior medical students from various regions and institutions of Saudi Arabia received training in POCUS, while 23.4% claimed to have received informal training. These numbers show slightly higher percentages than those conducted at King Saud bin Abdulaziz University for Health Sciences (KSAU-HS), an institution in Riyadh, that observed 21.4% of their 229 respondents receiving formal training, with 12.7% receiving informal training [12]. While 1.3% of their respondents have obtained accreditation in POCUS, 3.6% have obtained it in our multi-regional analysis. A gargantuan percentage of students (82.2%) have never used POCUS in our study; these numbers are a slight improvement over those reported by KSAU-HS, where 94.8% were never able to use POCUS, the majority of which (56.3%) cited an understaffed clinical setting. Our respondents mainly cited the lack of know-how (28.7%), with some feeling that it's not their place to perform it (24.8%).

Additionally, we evaluated the POCUS skills of senior medical students in perception and practice. Furthermore, we comparatively assessed these same parameters regarding physical examination. The more likely these students are to pick up certain diagnoses in physical examination, the more they see it applicable and practical to extrapolate these diagnoses on POCUS, ascites, thyroid masses, DVT, and hepatomegaly being the most likely diagnoses they'll achieve. In contrast, the highest deficiencies were noted in the diagnosis of Interstitial lung disease and cardiogenic shock. At KSAU-HS, they found hepatomegaly and intra-abdominal free fluid to be the simplest to evaluate, with POCUS and physical examination and abdominal aortic aneurysm being the most challenging.

With the data compiled from the senior medical students' experience, we can further understand certain variables that can impact their behavior and attitude towards POCUS through statistical correlations. Students who were given ultrasound courses in college were inclined to be POCUS self-learners. Interestingly, the perceived difficulty encouraged them to seek out information further and learn (Table 8). This further supports the need to develop and implement courses teaching POCUS. It can be surmised that there needs to be more access and knowledge when it comes to POCUS, especially with an astounding percentage of students (82%) who don't have access to it.

A worthwhile study was done at Harvard Medical School by Rempell et al. to integrate ultrasonography into the curriculum of first- and second-year medical students as part of their anatomical and diagnostic courses. One hundred seventy-six students were allocated into small groups with hands-on applications. Most of the students (91%) felt that the course was beneficial and believed it should be integrated into their fourth year as well [28]. This provides an excellent segue to a study conducted at the Brody School of Medicine at East Carolina University, United States. They developed a three-hour ultrasound teaching session for fourth-year medical students as part of their EM clerkship. Eighty-seven senior medical students participated in Focused Assessment with Sonography for Trauma (FAST), abdominal aorta, cardiac, biliary, renal, thoracic, and soft tissue/abscess imaging. In addition, students learned complex emergency ultrasound procedures, such as ophthalmic, advanced cardiac, DVT, testicular, and procedural applications. Most students strongly agreed that they would like to participate in more ultrasound sessions and ultrasound training sessions should be integrated into the curriculum [29].

On the grand scheme, a lot of progress has been made in the past decade; several medical schools worldwide have implemented or currently implement POCUS into their curricula. In the United States, 154 MD-granting medical schools' clinical ultrasound directors and curricula deans were surveyed in 2022. The survey had 25 questions about the characteristics of medical schools, barriers to implementing POCUS training to undergrad, and POCUS training as a stand-alone curriculum. More than one-half of medical schools who responded to the survey have already integrated POCUS into their undergraduate curricula [30].

The main limitation of our study was the poor response rate, which was expected due to the lengthiness of the survey, the small sample size, and the geographic distribution of medical schools in Saudi Arabia. More colleges with senior medical students were in the central region than others. Within the central region, response rates were highest from the capital city of Riyadh, Saudi Arabia, which has the largest population in the Kingdom.

Moreover, the survey distribution would be more efficient if done in person rather than remotely. However, since our study was conducted through all regions of Saudi Arabia, distributing the survey in person was not feasible. Additionally, if a shorter survey was conducted, it may have encouraged higher response and completion rates, albeit with less valuable/reportable results.

We want to encourage the implementation of POCUS in medical schools' curricula. This matter should be considered by medical education departments, the deans of medical schools, and postgraduate departments throughout the Kingdom and on the global stage. It could help medical students with their future medical careers and postgraduate training.

## Conclusions

POCUS is a utility and skill that can enrich the learning experience of undergraduate medical students. Considering that most participants have yet to take any medical school courses proposes a lack of awareness about its utility in the medical field. Nevertheless, many have shown keen interest in learning through courses, some even paying out of pocket to develop their skills. Additional courses with hands-on skills will improve the competence, confidence, and attitude of medical students with POCUS. It's also pertinent to raise awareness of POCUS among medical students regarding its implementations in many aspects of medicine and surgery, and this will guide them to seek knowledge through any platform to initiate their interest further.
